# miR-200c Regulation of Metastases in Ovarian Cancer: Potential Role in Epithelial and Mesenchymal Transition

**DOI:** 10.3389/fphar.2016.00271

**Published:** 2016-08-23

**Authors:** Siti A. Sulaiman, Nurul-Syakima Ab Mutalib, Rahman Jamal

**Affiliations:** UKM Medical Molecular Biology Institute, UKM Medical Centre, Universiti Kebangsaan MalaysiaKuala Lumpur, Malaysia

**Keywords:** miR-200c, ovarian cancer, metastasis, regulation, EMT

## Abstract

Among the gynecological malignancies, ovarian cancer is the most fatal due to its high mortality rate. Most of the identified cases are epithelial ovarian cancer (EOC) with five distinct subtypes: high-grade serous carcinoma, low-grade serous carcinoma, mucinous carcinoma, endometrioid carcinoma, and clear-cell carcinoma. Lack of an early diagnostic approach, high incidence of tumor relapse and the heterogenous characteristics between each EOC subtypes contribute to the difficulties in developing precise intervention and therapy for the patients. MicroRNAs (miRNAs) are single-stranded RNAs that have been shown to function as tumor suppressors or oncomiRs. The miR-200 family, especially miR-200c, has been shown to be implicated in the metastasis and invasion of ovarian carcinoma due to its functional regulation of epithelial-to-mesenchymal transition (EMT). This mini review is aimed to summarize the recent findings of the miR-200c functional role as well as its validated targets in the metastasis cascade of ovarian cancer, with a focus on EMT regulation. The potential of this miRNA in early diagnosis and its dual expression status are also discussed.

## Introduction

Ovarian cancer has the highest prevalence among the gynecological cancers worldwide with more than 238, 700 newly diagnosed cases and 151, 900 reported deaths per year (Ferlay et al., 2015; Siegel et al., 2016). Among the ovarian malignancies, 90% of the cases are epithelial ovarian cancer (EOC) with five identified subtypes namely, high-grade serous carcinoma (70%), low-grade serous carcinoma (<5%), mucinous carcinoma (3%), endometrioid carcinoma (10%), and clear-cell carcinoma (10%) (Prat, 2012b). The majority of these EOC categories are heterogeneous in nature, with distinct epidemiological and genetic factors, molecular profiles and behavioral responses toward chemotherapy and other treatments (Prat, 2012a; Groen et al., 2015), contributing to the difficulties in designing effective therapeutic strategies. Also, the lack of an early screening strategy (Buys et al., 2011) and the rather high rate of tumor relapse (Mei et al., 2013) increase the mortality rates in ovarian cancer patients. Therefore, it is necessary to develop more precise and effective treatment strategies in order to improve the survival rates for women diagnosed with this cancer.

MicroRNAs (miRNAs) have been recognized as a class of molecular regulators and potential therapeutic agents in cancers. miRNAs are short single-stranded non-coding RNAs (~21 nucleotides in length) which are able to negatively regulate gene expression by binding to complementary sites in the target messenger RNA (mRNA) at the 3′ untranslated regions (Fabian et al., 2010). Biosynthesis of miRNAs starts with the transcription of miRNAs in the nuclei (Lee et al., 2002; reviewed, Beezhold et al., 2010) and followed by the cleavage of miRNAs by Drosha complex into a distinctive stem-loop structure (Lee et al., 2003; reviewed, Beezhold et al., 2010). These miRNAs are exported to the cytoplasm, in which RNAse III enzyme, Dicer, cuts the miRNAs into single-stranded miRNAs (Ketting et al., 2001; reviewed, Beezhold et al., 2010). One strand of the miRNAs is integrated into Argonaute 2 protein complex to produce the RNA-induced silencing complex. A perfect complementary binding between miRNA and its mRNA target will lead to mRNA degradation, while ‘incomplete matching’ binding will lead to translational repression of the mRNA (Di Leva et al., 2006; Beezhold et al., 2010). Since one miRNA can target many genes (Royo et al., 2006), the evidence of miRNAs forming coordinated regulatory networks (Fazi and Nervi, 2008; Chowdhury et al., 2014) and act as oncomiRs or tumor suppressors may provide a novel function of these miRNAs as biomarkers for diagnosis or therapeutic purposes.

Several miRNAs have been shown to be implicated in ovarian cancers and one of them is the miR-200 family, which is abundantly expressed by epithelial tissues (Pal et al., 2015). The miR-200 family derives from two miR gene clusters; miR-200a, miR-200b, and miR-429 that are located at chromosome 1p33.36, and miR-200c and miR-141 that are located at chromosome 12p13.31 (**Table 1**) (Muralidhar and Barbolina, 2015). These miRNAs share a high degree of sequence homology (**Table 1**) with only one nucleotide difference at their seed sequence (Muralidhar and Barbolina, 2015), and they are usually categorized functionally by their seed sequence due to possible same target genes (Pecot et al., 2013). The Cancer Genome Atlas (TCGA) group performed genome analysis of 408 high-grade serous EOC (HGSOC) to identify molecular regulatory networks involved in EOC (The Cancer Genome Atlas Research Network, 2011). In this study (The Cancer Genome Atlas Research Network, 2011), they found that 89% of the affected genes were predicted to be regulated by eight main miRNAs including two members of miR-200 families which were miR-141 and miR-200a. Chen et al. (2013) systematically reviewed studies on miRNAs profiling of EOC and validated the findings from eight studies. They concluded that miR-141, miR-200a, miR-200b, and miR-200c were consistently upregulated only in four of the reported studies (Chen et al., 2013). One reason mentioned for such inconsistencies is that previous miRNAs profiling studies used various types of controls, and only a few did the comparison to normal ovarian epithelial cells, which is the most appropriate control (Zorn et al., 2003). A recent study performed on a larger EOC cohort (*n* = 100) and using ovarian epithelial cells as controls, confirmed that similar upregulation of these miRNAs was observed in EOC samples (Cao et al., 2014). Importantly, elevated expression of miR-200a and miR-200b was associated with advanced and high-grade tumors, while high miR-200c expression was only associated with advanced tumors (23). These diverse expression profiles of miR-200 family in different EOC progression and subtypes may suggest that miR-200’s regulatory role depends on the stage and histology profile of the ovarian cancer. Recent publications on the functions of miR-200 in ovarian cancer revealed that members of miR-200 family mainly act as metastases inhibitors (Iorio et al., 2007; Hu et al., 2009; Díaz-López et al., 2014; Kinose et al., 2014; Humphries and Yang, 2015; Muralidhar and Barbolina, 2015; Pal et al., 2015), however, there is still no differentiation of the roles of the individual members of the miR-200 family and how each of them regulate cancer progression especially in terms of ovarian cancer pathogenesis. Moreover, within the miR-200 family, miR-200c has also been shown to be a marker for epithelial-mesenchymal transition (EMT), which is the important step of cancer metastasis (Iorio et al., 2007; Hu et al., 2009; Díaz-López et al., 2014; Kinose et al., 2014; Humphries and Yang, 2015; Muralidhar and Barbolina, 2015; Pal et al., 2015). Therefore, this mini review will summarize the current knowledge on the downstream targets of miR-200 in ovarian cancer with an emphasis on the functional role of miR-200c in the metastasis cascade and discuss the prospective value and issues in early screening strategy for ovarian cancer.

**Table 1:** miR-200 members’ functional groups and seed sequences.

miR-200	Chromosome location	Mature seed sequence (5′–3′)	Functional group
miR-200a	1p33.36	UAACACUGUCUGGUAACGAUGU	Group I
miR-200b	1p33.36	UAAUACUGCCUGGUAAUGAUGA	Group II
miR-429	1p33.36	UAAACUGUCUGGUAAAACCGU	Group II
miR-200c	12p13.31	UAAUACUGCCGGGUAAUGAUGGA	Group II
miR-141	12p13.31	UAACACUGUCUGGUAAAGAUGG	Group I

*miR-200 family arises from two different gene clusters which are the chromosome 1p33.36 (miR-200a/b/429) and chromosome 12p13.31 (miR-200c/141). These miRNAs shares similar seed sequences (2–8 nucleotides) with a difference at 4th position (highlighted)*.

## miR-200c-Mediated Metastasis

**FIGURE 1:**
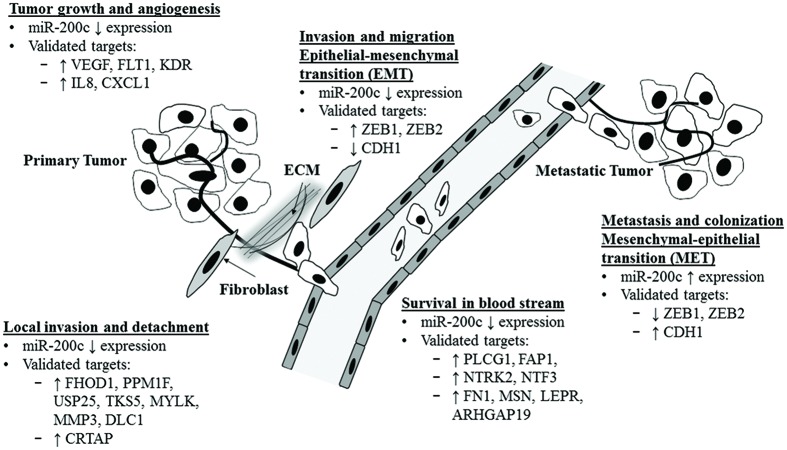
**Functional regulation of metastasis cascade by miR-200c and its validated targets.** Illustration of miR-200c functional role and its validated target in each of the metastases cascade. ECM, extracellular matrix.

Metastasis is defined as the multi-step processes of cancer cells colonization of distant organ or tissues (secondary/metastatic tumors) from the primary tumor site (Wan et al., 2013). Six main steps are involved namely: (1) tumor growth and angiogenesis at the primary site, (2) stromal invasion and detachment from extracellular matrix (ECM), (3) intravasation into the circulation, (4) survival in the circulation, (5) extravasation at secondary site, and (6) colonization of the new secondary site (Wan et al., 2013; Humphries and Yang, 2015). A recent review highlighted the role of the miR-200 family in each of the six steps (Humphries and Yang, 2015). **Figure 1** shows the summary of the actions of miR-200c action and its validated targets in each step discussed below.

### Metastasis Routes of the Ovarian Cancer Progression

Differently to other tumors, majority of EOC metastasize to adjacent sites along the peritoneum throughout the pelvic and abdominal cavity. The most common identified metastatic sites are including the pelvic wall, omentum and mesentery, though there are other adjacent metastatic sites have also been reported such as skin and lymph nodes (Cheng et al., 2009). Ovarian tumor cells spread through the peritoneal fluid or known as transcoelomic route of metastasis from the pelvic up to intestinal mesentery that allows for implantation of the tumors to different peritoneal organs (Meyers et al., 1987; Coakley and Hricak, 1999; Carmignani et al., 2003; Nougaret et al., 2012; Le, 2013). Though, this implantation of tumors depends on the fluidity of the peritoneum in which less fluidity will restrict the movement of the tumor cells at the primary site (Carmignani et al., 2003; Tan et al., 2006; Feki et al., 2009), thus may results in the accumulation of ascites in the abdomen. The formation of ascites is not well understood, yet previous studies have shown that ascites accumulation is regulated by VEGF (Kraft et al., 1999; Xu et al., 2000), a validated target of miR-200c, and ascetic fluid is primarily composed of tumor cells, white blood cells and lactate dehydrogenase (Runyon et al., 1988; Bansal et al., 1998; Feki et al., 2009; Kalogeraki et al., 2012). These floating tumor cells in ascites usually in multicellular spheroids (Lengyel, 2010) and implantation of these spheroids involves involves interactions between mesothelium and tumor cells within peritoneal cavity, thus may also interacting the adjacent areas including peritoneum, bowel, omentum and serosa (Daya and Elliott McCaughey, 1991; Kenny et al., 2007).

In rare occurrence, much further metastatic sites of the ovarian carcinoma have also been reported such as in the brain (Koul et al., 2001; Sekine et al., 2013; Longo et al., 2014), breast (Dursun et al., 2009; Karam et al., 2009; Abbas et al., 2013; Tempfer et al., 2016), lung (Carbonari et al., 2015; Upadhyay et al., 2015), pancreas (Nakamura et al., 2016), and gastrointestinal track (Zhou and Miao, 2012), thus suggesting an alternative route of metastasis in EOC. Previous studies have reported that the presence of circulating tumor cells (CTCs) in the blood circulation of the ovarian cancer patients (Pecot et al., 2011; Phillips et al., 2012), which implies that ovarian tumor cells may also follow ‘hematogenous’ route of metastasis through blood stream. In fact, Pradeep et al. (2014) showed that ovarian CTCs prefers hematogenous metastasis in a parabiosis animal model. In this study (Pradeep et al., 2014), the female mice had their skin removed from shoulder to the hip joint and then pairs of these female mice were surgically anastomosed. In each mice pairs, ovarian carcinoma cells (SKOV3ip1) were injected to abdominal cavity in one of the mice and these tumor cells metastasized to the omentum of the other mice (Pradeep et al., 2014). The immunofluorescent staining showed that both of the paired mice shared blood not lymphatic vessels (Pradeep et al., 2014) thus confirms the hematogenous route of circulation. This preferential hematogenous metastasis route is depends on the ERBB3/NGR1 signaling axis, whereby the suppression any of these molecular regulators, led to substantial reduction of the tumor metastasis (Pradeep et al., 2014). ERBB3, ERB-B2 tyrosine kinase 3 protein is a member of epidermal growth factor receptor (EGFR) family in which it binds to other kinases in EGFR family such as Neuregulin1, NGR1 and activates downstream signaling for cell proliferation and differentiation (Zhang K. et al., 2013; Reif et al., 2016). Moreover, recent study in mice also showed that ovarian tumor cells can hematogenously metastasize with the preference to the ovary (Coffman et al., 2016). This particular study (Coffman et al., 2016) is vital due to there are emerging findings that suggest that HGSOC originates from distant fallopian tube lesions and the progression of metastasis is to the ovary (Kuhn et al., 2010; Perets et al., 2013; Perets and Drapkin, 2016), thus may suggest the hematogenous metastasis may be the key for interventions in HGSOC. Nevertheless, there are about 40% of ovarian cancer patients did not have fallopian tube lesions and animal studies also showed that epithelial ovarian cells may also be the origins for HGSOC (Flesken-Nikitin et al., 2013; Zeppernick et al., 2015). Therefore, more works are needed to clarify the pattern for ovarian cancer metastasis via hematogenous route, and how it contributes to disease progression as well as patients’ survival outcomes.

### miR-200c-Mediated Tumor Growth and Angiogenesis

There is currently limited evidence to show the suppression of early tumor formation and growth by the miR-200 family, and much less for miR-200c. Two studies on miR-200b showed that the overexpression of miR-200b reduced tumor growth in prostate (Williams et al., 2013) and breast (Humphries et al., 2014) cancers. In contrast, another study reported that miR-200b did not exhibit any effect on the growth of breast cancer (Li X. et al., 2014). Since miR-200c has an identical seed region with miR-200b, miR-200c may have a similar regulatory function in tumor growth in the early step of metastasis. However, more investigations are needed to verify the functional roles of the miR-200 family, especially in ovarian cancer progression.

Early tumor development requires activation of angiogenesis which is the establishment of new blood vessels. A previous study on Ishikawa cells (endometrial adenocarcinoma) revealed that restoration of miR-200c expression resulted in reduced mRNA expression and protein level of vascular-endothelial-growth factor (*VEGF*) but only the protein level of *FLT1* (*VEGF* receptor 1; Panda et al., 2012). Both of these factors are important for angiogenesis. This suppression was attributed to the direct binding of miR-200c to the *VEGF* mRNA 3′UTR region but not with *FLT1* mRNA (Panda et al., 2012). A similar finding was also observed in lung cancer (Shi et al., 2013) in which miR-200c directly targeted *KDR* (*VEGF* receptor 2). These findings were also confirmed by a recent study in placenta tissues that showed miR-200c negatively regulated *VEGF* mRNA and protein levels (Hu et al., 2016), thus suggesting the regulation of angiogenesis by miR-200c. In a recent study on bone cancer, overexpression of miR-200c transformed the metastatic fast-growing tumor into a dormant tumor both *in vivo* and *ex vivo* and prolonged the survival rate of the mice injected with the tumors, due to the suppression of angiogenesis (Tiram et al., 2016). This further suggests that miR-200c negatively regulates angiogenesis and may provide beneficial outcomes in improving patient survival. In terms of ovarian cancer, overexpression of miR-200c in an ovarian cancer cell line (Hey8A) decreased *IL8* and *CXCL1* expressions and this suppression was reversed by the presence of miR-200c inhibitors (Pecot et al., 2013). *IL8* and *CXCL1* are also critical players of tumor vasculature and angiogenesis (Merritt et al., 2008; Tran et al., 2012; Pecot et al., 2013). Nevertheless, recent findings showed that miR-200b is a more prominent player in the regulation of angiogenesis compared to miR200c (Chan et al., 2011; Choi et al., 2011; Chang et al., 2013; Pecot et al., 2013), implying the specificity of these miRNAs despite having the same seed sequence. Therefore, more precise and larger studies are needed to differentiate their biological action and functional targets in regulating angiogenesis especially in ovarian cancer plus the implication on survival outcomes.

### miR-200c-Mediated Local Invasion and Detachment

Tumor invasion and migration involve the breaking of ECM and invasion to stromal tissues from primary tumor site before moving to the circulation. A study on the breast cancer cell line, MB-231, showed that miR-200c mimics reduced expression of formin homology domain-containing protein 1 (*FHOD1*) and Mg^2+^/Mn^2+^-dependent protein phosphatase 1F (*PPM1F*), in conjunction with reduced invasive capacities of these MB-231 cells (Jurmeister et al., 2012). *FHOD1* is an actin-nucleating formin that is primarily involved in the cytoskeletal rearrangement and is upregulated during EMT transition (Gardberg et al., 2013) while *PPM1F* kinase is involved in MAP kinase regulation of focal adhesion (Zhang S. et al., 2013). In lung cancer, miR-200c inhibits cancer cell invasion through the ubiquitin specific peptidase 25 (*USP25*) protein (Li J. et al., 2014), which is a peptidase primarily associated with protein ubiquitination and may intervene with TGFβ signaling and EMT (Soond and Chantry, 2011). A recent study on breast cancer to identify the functional targets of miR-200c revealed that among 12 validated targets, *CRTAP* was shown to be strongly implicated in cell invasion (Perdigao-Henriques et al., 2016). *CRTAP* is a cartilage-associated protein that is involved in the alteration of proline amino acid (proline 3-hydroxylation) during collagen formation (Hudson and Eyre, 2013). In the case of osteogenesis imperfecta, *CRTAP* expression is reduced in conjunction with the decrease in collagen binding of a proteoglycan, Decorin, that regulates TGFβ signaling in ECM (Grafe et al., 2014). Thus, tumor cells with decreased collagen binding properties may gain invasive properties due to changes in ECM properties and it will be useful to investigate the role of *CRTAP* in promoting invasion and metastases through TGFβ signaling in ovarian cancer. In another study on breast cancer, novel validated targets of miR-200c, SH3 and PX domains 2A (*TKS5*), and myosin light chain kinase (*MYLK*) were downregulated by miR-200c through the ZEB1 pathway (Sundararajan et al., 2015). *TKS5* is involved in the polymerization of cytoskeleton actin and ECM degradation (Murphy and Courtneidge, 2011), while *MYLK* is mainly involved in cytoskeletal rearrangement and muscle contraction (Dudek et al., 2002; Takashima, 2009), thus consistent with the reduced invasion and metastasis observed when these factors were suppressed by miR-200c (Sundararajan et al., 2015). A study on two ovarian cancer cell lines (OVCAR3 and SKOV3; Sun et al., 2014) showed that an overexpression of miR-200c decreased the expression and protein level of matrix metalloproteinase 3 (*MMP3*), which is the proteinase responsible to facilitate ECM breakdown, thus this led to inhibition of ovarian cancer cell invasiveness via ZEB1/pSMAD pathway, which further suggests the role of miR-200c in the molecular pathway of ovarian cancer invasion. Another validated target of miR-200c is *DLC1* (deleted in liver cancer-1), which is a tumor and metastasis suppressor that regulates actin formation and focal adhesion (Feng et al., 2013). In a study on EOC, forced expression of miR-200c reduced *DLC1* expression in conjunction with increased cell proliferation and suppression of serous EOC invasion and migration (Ibrahim et al., 2015), consistent with the known role of *DLC1*.

### miR-200c-Mediated Epithelial-Mesenchymal Transition (EMT)

Gain of cell motility is one of the vital steps in tumor migration and metastasis. One of the best described pathways (Pal et al., 2015) is the EMT which is the first indication of the cancer development. This EMT transition is categorized by a series of reversible processes of molecular and phenotypic alterations in the cells which leads to differentiation of EMT or mesenchymal to epithelial cells (MET). The epithelial tumor cells favor EMT to gain migration and invasive characteristics, however, when they reach a new secondary site, MET is activated to form macro metastasis (Craene and Berx, 2013; Nieto, 2013). This aberrant stimulation of multiple EMT/MET cycles and its flexibility contribute to the development of fibrosis and cancer metastases. Usually, this EMT/MET is marked by the loss of epithelial-cadherin protein, a type-I transmembrane protein (*CDH1*) which is an identity marker of epithelial cells (Yang and Weinberg, 2008).

Two key molecular targets in miRNA-mediated EMT pathway are the receptor-regulated SMADs (SMAD2 and SMAD3) and ZEB family proteins (ZEB1 and ZEB2). SMAD2 and SMAD3 proteins are the downstream effectors of TGFβ signaling pathway, whereby the activation of the TGFβ receptor leads to phosphorylation of SMAD2 and SMAD3 (Brown et al., 2007). Activated SMAD2 and SMAD3 will bind to SMAD4 and induce translocation to the nucleus, in which they will interact with different nuclear regulators and modulate transcription of target genes (Brown et al., 2007). ZEB1 and ZEB2 are the transcriptional repressors of *CDH1* gene (Gregory et al., 2008; Park et al., 2008; reviewed, Hill et al., 2013) and both contain two highly conserved zinc-finger domains (Muralidhar and Barbolina, 2015). This zinc-finger domain would allow DNA to be attached to the E-boxes in the promoter sections of a target gene (Muralidhar and Barbolina, 2015). Transcriptional suppression of *CDH1* gene is mediated by the binding of ZEB factors to the promoter of *CDH1* gene (Eger et al., 2005; Aigner et al., 2007). This loss of *CDH1* expression due to ZEB binding will result in migratory and invasive properties in cells with the appearance of spindle-shaped morphology (Vergara et al., 2010).

Hurteau et al. (2006) performed the repression of miR-200c expression using anti-miRs oligonucleotides in various cell lines and found that miR-200c expression was inversely correlated to the expression of *ZEB1* (Hurteau et al., 2006). A study on EMT transition by TGFβ in MDCK cells showed that expression of miR-200 members including miR-200c and *CDH1* were reduced, whereas *ZEB1* and *ZEB2* expression were elevated (Gregory et al., 2008). This functional relationship between *ZEB1* and miR-200c was further analyzed using a *ZEB1* knockdown in breast and colorectal cancer cell lines (Burk et al., 2008). In this study (Burk et al., 2008), a reduced *ZEB1* level led to significant overexpression of miR-200c and miR-141, and this was due to direct binding of *ZEB1* to their promoter regions (Burk et al., 2008). The elevated miR-200c expression led to MET transition in undifferentiated cancer cells with increased expression of *CDH1*. Since then, various studies have demonstrated that miR-200c and the ZEB family of transcription factors play major roles in the regulation of EMT and MET (Brabletz and Brabletz, 2010; Panda et al., 2012; Hill et al., 2013; Hur et al., 2013; Díaz-López et al., 2014; Koutsaki et al., 2014; Sundararajan et al., 2015; Jiao et al., 2016; Perdigao-Henriques et al., 2016).

Despite the translational suppression of *ZEB* by the miR-200 family, ZEB transcription factors can also regulate miR-200 gene expression by directly binding to the miR-200 promoter and suppress the expression (Hill et al., 2013). This negative double-feedback regulation between miR-200 and ZEB families provides greater flexibility in the regulation of cell fate. Normal epithelial cells express *CDH1* due to suppression of *ZEB1*/*ZEB2* due to high expression of miR-200 family, but changes in environment and signals can independently stimulate *ZEB1*/*ZEB2* expression which causes miR-200 family and *CDH1* expression to be repressed and allowing EMT to occur (Hill et al., 2013). Both of these ZEB regulators are implicated in miR-200-mediated repression of *CDH1* expression in epithelial cells (Gregory et al., 2008; Park et al., 2008; Ahn et al., 2012; Hill et al., 2013; Pal et al., 2015; Yang et al., 2015). Although miR-200c is known to repress *ZEB1*/*ZEB2* to restore *CDH1* expression in majority of the studies, there are reported findings that in some cancer cell lines, restoration of *CDH1* expression is due to miR-200c gain of expression but the migration and invasiveness properties in those cell lines were still reduced (Cochrane et al., 2010; Pacurari et al., 2013). These findings may imply that miR-200c regulation of cell motility is independent of *CDH1* and may also suggest miR-200c targets other molecular regulators and mechanisms besides ZEB1/CDH1 pathway. Moreover, the absence of *CDH1* gene expression in cancer can also imply that repression of *CDH1* in cancer may be caused by other mechanisms such as C/A single nucleotide polymorphism at the promoter of *CDH1* (Li et al., 2000; Wang et al., 2012) or due to *CDH1* promoter hyper methylation (Caldeira et al., 2006; Shargh et al., 2014), in which both can suppress *CDH1* expression.

Although substantial evidences show that miR-200c importantly regulates EMT/MET in ovarian cancer progression, lack of evidences on the transitions between epithelial to mesenchymal tumor cells especially in morphological features of mesenchymal-like cells in patients’ samples (Tarin, 2005; Ledford, 2011; Tsai and Yang, 2013) lead to dispute whether the transition between EMT to MET exists in the cancer progression and metastasis. An integrated genomic analysis of HGSOC done by TCGA network revealed that within the HGSOC, there are four mRNA transcriptional subtypes, including immunoreactive, differentiated, proliferative and mesenchymal, and three miRNA expressional subtypes that are overlapped with those four mRNA transcriptional subtypes (The Cancer Genome Atlas Research Network, 2011). Although, TCGA analyses did not show any difference of the survival time in the patients between those HGSOC molecular subtypes, another study have confirmed the presence of those HGSOC molecular subtypes and improved these molecular classifications with prognostic values (Konecny et al., 2014). In this study (Konecny et al., 2014), the immunoreactive subtypes HGSOC was identified with the best survival outcomes in the patients, while the mesenchymal and proliferative subtypes have the worst. Nevertheless, both of these studies showed that there can be more than one subtype in an individual at a time depending on the analyses and these findings may imply that the same tumor cells could also express multiple molecular profiles or miRNAs networks. Thus, further investigation was performed by Yang et al. (2013) to determine the miRNAs regulatory network in mesenchymal subtypes of HGSOC, which has the worst patients outcomes and they identified eight key miRNAs including two members of miR-200 families which were miR-141 and miR-200a that were shown to be associated with poor patients’ outcomes (Yang et al., 2013). However, in this study (Yang et al., 2013), miR-506 instead of miR-200c was shown to negatively regulate the EMT, cell proliferation, migration and invasion of ovarian tumor cells through a direct binding of the transcription factor, SNAI2 and subsequently suppressed *CDH1* expression. These findings show that regulation of *CDH1* expression and EMT by miR-506/SNAIL may be specific to mesenchymal subtypes, in contrast to epithelial subtypes of HGSOC, in which the miR-200c/ZEB may be the main regulators. Though, further research are needed to confirm these miRNA regulatory networks particularly that define the molecular subtypes of HGSOC. Nevertheless, none of these studies show any evidence of transition between the epithelial to mesenchymal subtypes nor mesenchymal to epithelial subtypes in HGSOC samples. Intriguingly, a study of gene expression profiling between primary and metastatic ovarian carcinoma revealed that despite no morphological difference between two tumor types, there were distinguishable gene clustering profiles that differentiate the metastatic cells from their primary cells (Lili et al., 2013) and these identified genes were mainly involved in EMT-related pathways. These findings may not demonstrate the existence of EMT/MET interconversion yet, they have shown that there are patterns of gene expressional changes from epithelial to mesenchymal genes, despite no morphological features are observed. Furthermore, recent study of various ovarian cancer types showed that expression of membrane *CDH1* was almost not detected in normal ovarian epithelium, but was higher in benign tumors, and progressively decreased in borderline or malignant tumors and in metastatic lesions (Wu et al., 2016). In this study (Wu et al., 2016), the expression of *VIMENTIN*, a marker for mesenchymal cells, was opposite to the *CDH1* expression, thus may support the EMT/MET dynamic interconversion during ovarian cancer progression. However, the role of CDH1 as a tumor suppressor in ovarian cancer does not follow the classical EMT profile (Auersperg et al., 1999; Martin et al., 2011; Rodriguez et al., 2012), due to up-regulation of *CDH1* expression observed in tumors that were derived from ovary (Auersperg et al., 1999), therefore more studies are needed to clarify the role of CDH1, miR-200c and other regulatory miRNAs, and their regulations of EMT/MET in ovarian cancer progression.

### miR-200c-Mediated Tumor Intravasation

Intravasation describes the invasion of the cancer cells into the blood stream or lymphatic system. This mainly involves the Notch signaling (Sonoshita et al., 2011) and TNF-α/tumor-associated macrophages (Wyckoff et al., 2007; Zervantonakis et al., 2012; Roh-Johnson et al., 2014). How exactly the miR-200 family regulates intravasation is largely unknown, although in a study on breast cancer found that both expression of miR-200 gene clusters and overexpression of *CDH1* can independently inhibit the intravasation into the blood stream (Korpal et al., 2011). However, limited research have been done to examine the role of miR-200 family in cancer cell intravasation and this is due to difficulties in measuring intravasated cells (Humphries and Yang, 2015), therefore future work should be done in focusing on this area.

### miR-200c-Mediated Tumor Survival and Circulation

Intravasated cells in the blood stream or lymph nodes are also known as the circulating cancer cells (CTCs) and these CTCs can re-program cellular processes such as apoptosis and anoikis to enhance their survival rate (Humphries and Yang, 2015). In the MDA-MB-231 breast cancer cell line, overexpression of miR-200b/c/429 gene cluster as compared to miR-200a/141 caused increased apoptosis, increased caspase activity and reduced cell viability through phospholipase C gamma 1 (*PLCG1*; Uhlmann et al., 2010). In a study on ovarian cancer, miR-200c expression led to suppression of Fas-associated phosphatase-1 (*FAP1*) and increased *CD95*, a death receptor, surface expression and consequently led to increased cell sensitivity to CD95-mediated apoptosis during CD95 agonist treatment (Schickel et al., 2010), therefore restoring the tumor cells sensitivity toward CD95-mediated apoptosis. Incomplete attachment of cells to ECM will induce anoikis (Humphries and Yang, 2015), which is another form of apoptosis, and surviving this process is one of the critical steps in cancer cell progression. Although the mechanisms by which the cancer cells evade anoikis are unknown, the gain of miR-200c expression in endometrial and breast cancer in cultures restored the sensitivity of these cells toward anoikis, through suppression of neurotrophic-tyrosine-receptor kinase type 2, *NTRK2* (Howe et al., 2011). In this study (Howe et al., 2011), miR-200c also suppressed the expression of fibronectin 1 (*FN1*), moesin (*MSN*), leptin receptor (*LEPR*) and Rho-GTPase-activating protein 19 (*ARHGAP19*), and consequently inhibited migration of these cells. A study of *NTRK2* and anoikis in breast cancer cell lines also showed that miR-200c can directly target neurotrophin 3 (*NTF3*) and this NTRK2/NTF3 signaling is the downstream effector of NFKB signaling which promotes anoikis (Howe et al., 2012). In ovarian cancer cell lines, *NTRK2* was shown to suppress anoikis through the PIK3-AKT pathway that resulted in more invasive and chemo-resistant cells (Yu et al., 2008), thus suggesting the novel function of miR-200c in anoikis besides EMT/MET cycles. In contrast, miR-200a was shown to promote anoikis resistance via its target, yes-associated protein 1, *YAP1* (Yu et al., 2013). The functional discrepancies in miR-200 family may be due to the single-nucleotide differences in the seed sequence thus allowing opposite downstream actions. However, further research is needed to elucidate the role of each member of miR-200 in anoikis, and also their interaction with immune system as these cancer cells managed to escape immune surveillance to metastasize.

### miR-200c-Mediated Tumor Extravasation and Colonization

Circulating tumor cells which survived will colonize their new niche by extravasating from circulation, induce cell proliferation and inhabit the new area. In order for these CTCs to adhere to endothelial cells in the new niche, they undergo MET to facilitate the settlement by recovering their epithelial properties. In contrast to the suppression of EMT by miR-200 family, this metastatic colonization of cancer cells are shown to be promoted by the miR-200 family (Drake et al., 2009; Korpal et al., 2011). In a study using the mouse mammary tumor cell lines, loss of miR-200b/c/429 cluster expression was observed in the 4TO7 cells that did not colonized distant organ efficiently when compared to the strongly metastatic 4TI cells (Dykxhoorn et al., 2009). Importantly, when this 4TO7 cells gained miR-200c/141 gene cluster expression and were then injected in the mice, 80% of those mice developed lung metastases (Dykxhoorn et al., 2009). Similarly in another study (Korpal et al., 2011), stable expression of either gene clusters of miR-200 (miR-200a/b/429 and miR-200c/141) in 4TO7 cells also resulted in lung and liver metastases when these cells were injected in mice. Moreover, in this study (Korpal et al., 2011), the miR-200c/141 gene cluster was more potent in promoting metastasis, and this miR-200-mediated metastases was independent of *CDH1*, thus suggesting other molecular target or pathway that mediates this metastasis process. Similar observation was also seen in a miRNAs profiling study of human breast cancer primary tumor in comparison to metastatic tumors (Gravgaard et al., 2012). Therefore, these studies suggest that all members of miR-200 suppress cancer progression by targeting the early steps in metastases cascade, however, the miR-200c/141 gene cluster may promote the advanced stage of metastases hence may explain the dual expression of miR-200c in ovarian cancer (Bendoraite et al., 2010).

## miR-200c Potentials in Prognosis and Early Diagnosis for Ovarian Cancer

### Circulating miRNAs in the Blood

Many studies have shown that miRNAs exist in the circulating blood which represents the environment of tissues or organs. MicroRNAs have also been shown to be released into the blood and are internalized into adjacent target cells (Chen et al., 2015). Circulating miRNAs are stable even in harsh conditions such as boiling and repetitive freeze-thaw cycles due to their association with proteins (Mitchell et al., 2008; Arroyo et al., 2011; Cortez et al., 2012), thus making miRNAs as the most suitable candidate for ovarian cancer early diagnostic screening. A study of sera collected from 28 serous EOC patients and compared to 28 healthy normal controls (Kan et al., 2012), showed elevated expression of miR-200a, miR-200b, and miR-200c in cancer patients with miR-200c expression being most significantly altered. Another study using sera collected from 70 EOC patients and compared to 70 age-matched healthy controls revealed elevated expression of miR-200c in all advanced stage EOC patients, and in all 18 EOC patients with confirmed metastases (Zuberi et al., 2015). These findings showed the possibility of using miR-200c as a diagnostic tool for ovarian cancer through the fluid biopsy approach. It is still necessary to investigate the role of miR-200c in the prognosis of ovarian cancer in a much larger cohort to evaluate the specificity of the miRNAs action in different EOC stages and subtypes.

### Circulating Exosomal miRNAs

Another source of circulating miRNAs is from exosomes, which are small membrane vesicles (30–100 nm in size) that are secreted by different cells types such as dendritic cells, lymphocytes and also tumor cells by exocytosis (Srivastava et al., 2015; Yu et al., 2015). Exosomes exist in almost all body fluids including plasma, breast milk, and saliva (Lässer et al., 2011; Yu et al., 2015) and commonly contains proteins, lipids, and nucleic acids such as miRNAs. The packaging of miRNAs in exosomes is selective and usually depends on their cell origin (Pant et al., 2012; Schwarzenbach, 2015). Exosomes are involved in cell-to-cell communication by transferring proteins, lipids, and nucleic acids that may also result in the change of wild type cells to malignant cells (Ratajczak et al., 2006; Valadi et al., 2007; Chen et al., 2012). In a study on ovarian cancer, miRNAs of tumor-derived exosomes were isolated from 50 serous ovarian carcinoma patients’ sera (110). Interestingly, the list of differentially expressed miRNAs is similar to another study performed on tissues (Iorio et al., 2007), suggesting that isolated miRNAs of tumor-derived exosomes may sufficiently reflects the expressional miRNAs profiles of tumor tissues. In this exosome study (Taylor and Gercel-Taylor, 2008), the expression of eight miRNAs (miR-21, miR-141, miR-200a, miR-200b, miR-200c, miR-203, miR-205, and miR-214) were upregulated while none of these circulating miRNAs were detected in the normal healthy samples. Notably, miR-200c and miR-214 expression were reduced in stage I patients in comparison to stage II and III patients, though across all samples, their expression were higher in all malignant groups when compared to benign and healthy groups (Taylor and Gercel-Taylor, 2008), which may indicate the variability of miRNAs expression derived from exosomes as well as due to the tumor stage and progression. A more recent study involving 163 EOC patients revealed a similar finding of elevated miR-200 family in ovarian cancer patients compared to normal samples (Meng et al., 2016). The study also found that miR-200c and 200b were associated with lymph node metastasis, FIGO stage III-IV and poor survival outcomes, while miR-200a was implicated with all stages. These differential elevated miRNAs may be due to disease progression especially in different histological subtypes between the EOC samples, or may also be due to selective packing of exosomes in which the exosomes respond to different types of miRNAs to facilitate the changes. Nevertheless, these newly identified miRNAs in exosomes may become a new non-surgical diagnostic target for early screening purposes.

### Circulating miR-200c Potential in Early Diagnosis

A recent systemic review reported the potential role of miR-200c as a biomarker for diagnosis in ovarian cancer (Shao et al., 2015). No relationship was found between miR-200c expression in the tissue samples with the patients’ outcomes (12 out 18 studies), however, when the tissue samples were categorized into the histological subtypes and cancer stages (four out of 12 studies), low expression of miR-200c in the primary tissue was negatively correlated with poor outcome but only in stage I patients (Shao et al., 2015). Since miR-200c suppresses EMT through suppression of ZEB1/2, early stage tumors with reduced miR-200c expression may gain migratory and invasive properties following EMT resulting in a greater chance for metastases, leading to poor prognosis of patients. Only six studies analyzed the serum/blood miR-200c levels in cancer patients (Shao et al., 2015), and five out of these six studies showed that high levels of miR-200c in the sera correlated with the worst outcomes in the patients irrespective of the cancer stages. Three studies involving patients with advanced stage ovarian also revealed the similar relationship between miR-200c expression and survival outcomes (Shao et al., 2015). The increase of miR-200c in the advanced stages may suggest MET in the cancer cells to drive the settlement in the new niche. Similarly, in studies on colorectal and breast cancers, miR-200c expression in the sera correlated positively with metastatic tissues, but not with primary tumor tissues (Hur et al., 2013; Toiyama et al., 2014; Madhavan et al., 2016), indicating the dynamic change of miR-200c expression at different points of cancer progression. This specificity of miR-200c expression pattern between tissue and blood samples may offer the potential of miR-200c as a biomarker in ovarian cancer to distinguish between early and advanced tumors. There is a need for larger studies to assess the functional outcomes of miR-200c in ovarian cancer patients, as well as determining the differences among the subtypes in terms of cancer progression for developing effective intervention and improving survival outcomes.

### miR-200c Dual Expression Profile in Ovarian Cancer Treatment

miR-200c has a prominent role in the regulation of EMT/MET transition and its expression levels in EOC patients are influenced by the subtypes and grades of the cancer (Bendoraite et al., 2010). One of the key issues with the treatment of ovarian cancer is the high incidence of relapse, which is mediated by the presence of chemotherapy resistance. One of the mechanisms that contributes to chemotherapy resistance in ovarian cancer is through *TUBB3*, a class III β-Tubulin (Umezu et al., 2008; Aoki et al., 2009; Roque et al., 2013). miR-200c has been shown to directly target *TUBB3* mRNA and repress its expression, thus increasing the chemo-sensitivity to drugs such as paclitaxel and docetaxel (Cochrane et al., 2010; Howe et al., 2011; Chang et al., 2015). Subsequently, low expression of miR-200c is implicated in poor patients’ outcome and paclitaxel resistance in ovarian cancer (Leskelä et al., 2011; Marchini et al., 2011). The overexpression of miR-200c led to suppression of *TUBB3* and restored the paclitaxel sensitivity in chemotherapy-resistant cancer cell lines (Cochrane et al., 2009, 2010; Cittelly et al., 2012) and in a xenograft tumor model (Cittelly et al., 2012), suggesting the positive role of miR-200-c as therapeutic agents in ovarian cancer treatment. There is a study which showed that a high expression of miR-200c was also associated with poor prognosis in serous ovarian cancer (Nam et al., 2008), while another showed no association between miR-200c expression and the patients’ outcome (Hiroki et al., 2010). These differences in outcomes linked to the role of miR-200c in chemotherapy may illustrate the underlying mechanisms in the miR-200c regulation of *TUBB3*. This theory is supported by the presence of the RNA binding protein Hu-antigen R (*ELAVL1*), which stabilizes *TUBB3* and promotes its abundance (Raspaglio et al., 2010), and can cooperate with miRNAs to repress mRNA expression (Wang et al., 2013; Kotta-Loizou et al., 2014). Depending on the location of the *ELAVL1* in the cells, miR-200c exerts differential actions on *TUBB3* expression. When the localization of *ELAVL1* is predominantly in the nucleus, miR-200c represses expression of *TUBB3* leading to sensitivity to chemotherapy, while cytoplasmic *ELAVL1* interacts with miR-200c to enhance expression of *TUBB3* and causes chemotherapy resistance and poor outcomes (Prislei et al., 2013). These differential associations between *TUBB3*, *ELAVL1*, and miR-200c may clarify the differences in the chemotherapy treatment outcomes particularly in the action of miR-200c in ovarian cancer. Other studies also have shown that cytoplasmic *ELAVL1* expression correlated with poor survival outcomes (Erkinheimo et al., 2003; Denkert et al., 2004) and the nuclear *ELAVL1* expression correlated with better outcomes in the patients (Yi et al., 2009). Nevertheless, a recent study done to determine prognostic role of *ELAVL1* in ovarian cancer found no significant role of *ELAVL1* localization in determining the patient outcomes as well as its interaction with miR-200c (Davidson et al., 2016). These inconsistencies in the findings may due to the technical aspects of those studies, such as the various stages of the EOC progression, in which the recent study only analyzed the late stages of serous EOC as compared to the others which involved a wider range of samples. Moreover, the prognostic roles of miR-200c and TUBB3 in restoring chemotherapy sensitivity in ovarian cancer is possibly a cell-context dependent (Brozovic et al., 2015). Therefore, further research are needed especially in animal models to standardize the stages of cancer progression as well the sites of the samples taken in order to determine the complexity and interaction of *ELAVL1* and miR-200c in regulating *TUBB3* expression and contribution to chemotherapy resistance and patient outcomes in ovarian cancer.

In terms of platinum-based chemotherapy drugs such as cisplatin, oxaliplatin, and carboplatin, the main mechanism for these drugs is to damage the DNA and thus induces genetic instability within the tumor cells. Previous studies have shown that the platinum-based drug resistance in ovarian cancer treatment primarily due to the sequestering of the drugs in the sub-cellular compartments in the cells via ATP-dependent Cu^+^ transporting P-type 7A or 7B (ATP7A/ATP7B) (Samimi et al., 2004; Yoshizawa et al., 2007; Kalayda et al., 2008; Safaei et al., 2012, 2013; Harrach and Ciarimboli, 2015) or due to ATP7A/ATP7B chaperone protein, ATOX1 (Safaei et al., 2009). Recent study has shown that other transporters such as LRRC8A, cation transporter (Sørensen et al., 2016) can also contribute to the cisplatin resistance. Nevertheless, until now, there is no available research that has shown the validated relationship between the platinum-based drug resistance and miR-200c expression and their consequences in the ovarian cancer patient survival, nor any of the research directly shows the molecular targets of miR-200c being implicated in the resistance in ovarian cancer. However, in a study in esophageal cancer, an expression of a subunit phosphatase 2A (*PPP2R1B*) that negatively regulates AKT phosphorylation was increased following knockdown of miR-200c and this protein was implicated in the resistance to cisplatin (Hamano et al., 2011). In a study of melanoma (Liu et al., 2012), a knockdown of a polycomb group protein (*BMI1*) expression in these tumor cells resulted in higher chemo sensitivity of these cells toward cisplatin together with a presence of reduced ATP-binding-cassette (ABC) transporters, which are the transporters of various substance in the cells. BMI1 is a validated target of miR-200c (Cui et al., 2014), thus this suppression of *BMI1* by miR-200c may therefore also suppresses ABC transporters levels and promotes the cisplatin sensitivity in ovarian cancer, however, further research is needed to address this relationship and how will it affects the survival outcomes of the ovarian cancer patients. Nevertheless, the majority of the findings showed the potential of miR-200c as a biomarker for early screening and the possible therapeutic approach for women with ovarian cancer has more credibility and potential than its chemo-resistance role.

## Conclusion

The lack of early screening strategy in ovarian cancer as well as the incidence of chemo-resistance and relapse during treatment contribute to the high mortality rate of this disease. Extensive studies have been done to identify the factors regulating the metastasis cascade in cancer progression. MiRNAs have been shown as potential regulators, with possible value as a biomarker and also as a therapeutic agent, particularly the miR-200c. Despite the reported issue of chemo-resistance with elevated expression of miR-200c in ovarian cancer patients, it seemed that miR-200c provides more beneficial outcomes as a biomarker and possibly as therapeutic agents due to its functional regulation of EMT and metastasis. Future research should be done to determine the role of individual miR-200 family members in regulating the metastasis and cancer progression, especially in a large cohort to further clarify the molecular network and pathway of miR-200c regulation. There is also a need to investigate the validated targets of miR-200c in animal models to differentiate their function between EOC subtypes and grades as well as their impact on survival outcomes.

## Author Contributions

SS drafted and wrote this manuscript, N-SM and RJ were responsible for idea conception, critical evaluation, and manuscript review.

## Conflict of Interest Statement

The authors declare that the research was conducted in the absence of any commercial or financial relationships that could be construed as a potential conflict of interest.
